# Effects of add-on montelukast on airway hyperresponsiveness in patients with well-controlled asthma – a pilot study

**DOI:** 10.3109/21556660.2013.791300

**Published:** 2013-04-02

**Authors:** Nina Kononowa, Sandra Michel, David Miedinger, Christiane E. Pichler, Prashant N. Chhajed, Arthur Helbling, Jörg D. Leuppi

**Affiliations:** 1 Clinic of Internal Medicine, University Hospital BaselSwitzerland; 2 Clinic of Allergology, University Hospital Basel, Switzerland; University Clinic of Rheumatology, Clinical Immunology and Allergology, University Hospital BernSwitzerland; 3 Medical Faculty, University of Basel and Clinic of Internal Medicine, Canton Hospital Baselland, LiestalSwitzerland; 4 University Clinic of Rheumatology, Clinical Immunology and Allergology, University Hospital BernSwitzerland

**Keywords:** Airway inflammation, Airway hyperresponsiveness, Asthma, Asthma control, Mannitol provocation test, Methacholine provocation test, Leukotriene receptor antagonists, AHR, Airway hyperresponsiveness; AQLQ, Asthma Quality of Life Questionnaire; BHR, Bronchial hyperreactivity; BPT, Bronchial provocation test; ECP, Eosinophil cationic protein; FeNO, Fraction of exhaled nitric oxide; FEV_1_, Forced expiratory volume in one second; ICS, Inhaled corticosteroids; LABA, Long-acting beta-adrenoreceptor agonists; LTRA, Leukotriene receptor antagonist; PD15 FEV1, resp. PD20 FEV_1_, Mannitol or methacholine dose leading to reduction in FEV_1_ during a bronchial provocation test, ≥15% or ≥20%, respectively; RDR, Response dose ratio is the percent decrease in FEV_1_ at the end of challenge divided by the cumulative dose of mannitol or methacholine causing decrease in FEV_1_

## Abstract

**Objective:**

Control of airway inflammation is the cornerstone of asthma management. The aim of the present pilot study was to assess the effects of a leukotriene receptor antagonist (LTRA) added to a basic treatment of inhaled corticosteroids (ICS) and long-acting beta-agonist (LABA) on airway hyperresponsiveness, inflammation, and quality of life in well-controlled patients with asthma.

**Research design and methods:**

Seventeen patients (age 18–65, 11 women) with well-controlled asthma presenting airway hyperresponsiveness to mannitol and methacholine challenge were given add-on montelukast on a stable ICS + LABA for 4 weeks. Quality of life and selected parameters of airway inflammation were measured at baseline and at study end. (ClinicalTrials.gov (NCT01725360)).

**Results:**

Adding montelukast to ICS + LABA resulted in an increase in mean FEV_1_ (+4.5%, *p* = 0.057), cumulated higher dose of mannitol (+32.5%, *p* = 0.023) and methacholine (+17.2%, 0.237) in the provocation test, lower airway reactivity with mannitol and methacholine (response dose ratio (RDR) –50.0%, *p* = 0.024 and –44.3%, *p* = 0.006, respectively), and improved airway sensitivity to mannitol and methacholine (+12.1%, *p* = 0.590 and +48.0%, *p* = 0.129 for PD15 and PD20 FEV_1_, respectively). Changes in inflammation parameters (blood eosinophil count, serum eosinophil cationic protein, and exhaled nitric oxide) were consistent with these findings. Asthma-related quality of life improved significantly in all domains and overall (from 5.3 at baseline to 6.1 at the final visit, *p* < 0.001). The main limitation was the absence of a control group.

**Conclusion:**

The consistency of the changes in airway hyperresponsiveness and inflammation as well as in quality of life observed with an add-on therapy with montelukast in well-controlled asthma patients during 4 weeks suggests that residual inflammation may represent an area for further improvement of asthma control to be explored in adequately powered randomized controlled trials.

## Introduction

Asthma is a chronic inflammatory disorder of the airways in which chronic inflammation is associated with airway hyperresponsiveness that leads to recurrent episodes of wheezing, breathlessness, chest tightness, and coughing^1^. Thus, control of airway inflammation is considered the cornerstone of asthma management^1^. Inhaled corticosteroids (ICS) are effective anti-inflammatory agents widely used for improving asthma control^1^. However, ICS may not suppress all components of airway inflammation^2,3^. Oral leukotriene receptor antagonists (LTRAs) have been shown to possess anti-inflammatory properties complementary to ICS^4,5^ and may reach small airways not easily accessed by inhaled formulations^6^. LTRAs have also been shown to attenuate airway hyperresponsiveness to both specific and non-specific stimuli^7,8^ and, as an add-on therapy to ICS, to improve asthma control^9–12^. When added to ICS, LTRAs may primarily have anti-eosinophilic effects^13,14^ and/or modulate airway hyperresponsiveness^15,16^.

Airway sensitivity to a challenge test is expressed as the cumulated provocative dose (in mg) of either methacholine or mannitol that induces a predefined and substance-specific percentage (20% and 15%, respectively) of the fall in FEV_1_ from baseline (PD20 and PD15, respectively). Airway reactivity is characterized by the response dose ratio (RDR), i.e. the final % fall in FEV_1_ divided by the total cumulative dose needed to induce that % fall in FEV_1_^17^. The assessment of airway reactivity by challenge tests is well-established and recommended in the Global Initiative for Asthma (GINA) guidelines for establishing the diagnosis of asthma since 2007^1,17^.

The aim of the present pilot study was to evaluate the effects of add-on montelukast to standardized ICS + LABA therapy on airway hyperresponsiveness, selected markers of airway inflammation, and quality of life in a cohort of already clinically well-controlled patients with asthma.

## Patients and methods

All patients were recruited at the outpatient clinic of Allergology and Clinical Immunology of the University Hospital of Bern, Switzerland. Seventeen adult patients older than 18 years and younger than 65 years of age with clinically well-controlled asthma under a fixed combination of budesonide/formoterol (or equivalent) were included after having signed an informed consent form. Clinically well-controlled asthma was defined as the absence of symptoms and exacerbations during the 8 weeks prior to inclusion. In addition, documented airway hyperresponsiveness to mannitol and methacholine as well as current non-smoker status were required for inclusion. Smokers, women planning a pregnancy or pregnant, and patients with any significant illness that could interfere with the feasibility or the results of the provocation tests, such as acute asthma or severe cardiovascular disease, were not included.

At baseline, all patients underwent a mannitol provocation test and the fraction of exhaled nitric oxide (FeNO) was measured. If positive, and at least 2 days later, a methacholine provocation test and repeated measurement of FeNO were performed. For standardization all patients were switched to the same dose-equivalent ICS + LABA therapy in a fixed combination with budesonide/formoterol (200/6 µgbid). After a 2-week run-in period on this therapy, 10 mg montelukast was added for a 4 weeks’ treatment duration (interim visit). All patients included attended the final visit in which the provocation tests were repeated.

FEV_1_ was assessed by using a MasterScreen Pneumo spirometer (Jaeger GmbH, Würzburg, Germany) with filter and according to ATS recommendations^18^. The best of three values FEV_1_ repeatable to within 100 ml were recorded and the percentage of predicted values^19^ were calculated. Mannitol challenge tests were carried out using the protocol described by Anderson *et al*.^20^. Mannitol was administered as a dry powder in capsule form, inhaled from a RS01 device (Pharmaxis Ltd. French’s Forrest, NSW, Australia [Trimedal AG, Brüttisellen, Switzerland] by [Stallergenes AG, Dietikon, Switzerland]). An empty capsule was used as a control at baseline. The test was started with 5 mg and increased doubling the doses up to 160 mg. In case of no response at the 160 mg dose, this dose was repeated up to a maximum cumulative dose of 635 mg. FEV_1_ was measured 60 seconds after delivery of each dose. The challenge was stopped if FEV_1_ fell by 15% or more, or when the maximum dose had been administered. The dose causing a 15% fall in FEV_1_ (PD15 FEV_1_) was read by interpolation on the plotted dose–response curve. Methacholine testing was carried out according to the protocol of the SAPALDIA Study^21^ which was identical to the protocol of the European Community Respiratory Health Survey (ECRHS) “method 2 short-protocol”^22,23^. The provocation test started with inhalation of saline diluent, and the maximum post-diluent FEV_1_ recorded 2 minutes later was the control value. All solutions of methacholine were prepared by the pharmacy of University Hospital Bern. Methacholine was delivered using a Mefar MB3 dosimeter (Mefar, Bovezzo, Italy) set to deliver the aerosol over a period of 1 second. FEV_1_ was recorded 2 minutes later and in the absence of a 20% fall in FEV_1_ from baseline the next dose was given. Methacholine was inhaled with quadrupling doses until a fall in FEV_1_ greater than 20% from the control value was observed or the maximum cumulative dose of 5.515 mg was reached. The dose causing a 20% fall in FEV_1_ (PD20 FEV_1_) was read by interpolation on the plotted dose–response curve. Patients were asked to refrain from short-acting beta-2-agonist for 8 hours, from the combined inhaled therapy (formoterol and budesonide) for 24 hours and from montelukast for 4 days prior to bronchial challenge testing.

Exhaled nitric oxide was measured with the same equipment throughout the study according to ATS guidelines^24^ by using an NIOX nitric oxide analyzer with computed biofeedback software by Aerocrine AB, Solna, Sweden.

Asthma-related quality of life (AQLQ) was assessed by applying the self-administered Juniper questionnaire at the baseline, interim and final visit. This validated questionnaire encompasses 32 items clustered in four domains: asthma symptoms, limitations in daily physical activities, emotional function, and exposure to environmental stimuli^25^. Each item was rated by the patient on a numerical scale ranging from 1 (extreme limitations) to 7 (no limitation at all).

Blood samples were drawn at the baseline and final visit before any challenge tests, for determination of blood eosinophils and eosinophil cationic protein serum levels (ECP, Pharmacia diagnostics, Uppsala, Sweden, now Fisher Scientific).

Predefined parameters of interest were the changes in PD15 FEV_1_ in the mannitol test, PD20 FEV_1_ in the methacholine test, the RDR for mannitol and methacholine, the asthma related quality of life, FeNO, blood eosinophils and eosinophil cationic protein concentrations between baseline and the final visit. Descriptive statistical analysis methods were used. No PD15 or PD20 value was assigned to subjects who did not achieve PD15 or PD20. As all parameters were normally distributed, means, standard deviations and corresponding 95% confidence intervals were calculated. For mean PD15 FEV_1_, PD20 FEV_1_, RDR, and FeNO the base 10 antilog (the antilog of the mean of log-transformed data corresponds to the geometric mean) was calculated as previously suggested^26,27^. Calculations of statistical significance (*p*-value) and corresponding power for a 5% significance level were performed by Student’s *t*-test using StatsDirect software version 2.7.7, Altrincham, Cheshire, UK.

The study was approved by the ethics committee of the University Hospital of Bern (KEK-BE 240/06), Switzerland. The study is registered with ClinicalTrials.gov (NCT01725360).

## Results

Seventeen patients (11 women, 65%), with a mean age of 32 (±11) years were included with baseline characteristics shown in Table 1. Mean FEV_1_ (3.35 L) and Tiffeneau index (74% of vital capacity) were consistent with patients with normal lung function, as required by the inclusion criteria. While mean blood eosinophil counts were within normal range, mean serum ECP levels were slightly above. Asthma-related quality of life was high at baseline, scoring 5.3 on a numerical scale up to 7.

**Table 1.** Baseline characteristics of asthma patients included in the pilot study. Mean values.

Number of patients included	17
Female sex (%)	11 (65%)
Age (years) ± SD	32.0 ± 11.0
FEV_1_ (L) ± SD	3.35 ± 0.84
Tiffeneau index (FEV_1_ as % of vital capacity) ± SD	73.8 ± 7.9
Serum eosinophils (G/L, normal range 0.02–0.40) ± SD	0.26 ± 0.21
Serum eosinophil cationic protein (µg/L; normal range 1.0–16.0) ± SD	16.9 ± 8.8
Mean asthma-related quality of life according to Juniper (numerical scale from 1, severely impaired, to 7, not impaired at all)* ± SD	5.3 ± 1.1

Mean ± standard deviation. FEV_1_: forced expiratory volume in 1 second.

As given in Table 2, 4 weeks of oral treatment with montelukast added to a backbone therapy with a fixed combination of ICS + LABA in well-controlled asthma patients presenting with an airway hyperresponsiveness to mannitol challenge, lead to a modest but consistent improvement of all parameters compared to baseline: numerically higher mean FEV_1_ values (+4.5%, *p* = 0.057), significantly higher mean dose of mannitol that could be administered during the provocation test (+32.5%, *p* = 0.023), lower sensitivity to mannitol as suggested by decreasing PD15 FEV_1_ values (+9.2%, *p* = 0.989), and lower bronchial reactivity as suggested by a significantly lower maximal percent decrease in FEV_1_ (−36.4%, *p* = 0.027) and by a decreasing RDR (−76.9%, *p* = 0.099 and −50%, *p* = 0.024 after logarithmic transformation). Fourteen out of 17 patients had no PD15 to mannitol at the end of the study. As shown in Table 3, methacholine BPT lead to a numerical improvement of all parameters of lung function (higher FEV_1_, higher cumulated methacholine dose, lower maximal % decrease in FEV_1_, improved airway sensitivity) and to a significantly decreased airway reactivity (RDR −48.1%, *p* = 0.01 and −44.3%, *p* = 0.006 after logarithmic transformation). Nine out of 17 patients had no PD20 to methacholine at the end of the study.

**Table 2.** Changes in mannitol test. Mean values ± SD (95% confidence interval).

	Baseline visit	Interim visit (+2 weeks)	Final visit (+6 weeks)	% change final vs. baseline (*p*-value)	Power for 5% significance
FEV_1_ (L)	3.350 ± 0.837 (2.920–3.780)	3.634 ± 1.057 (3.090–4.177)	3.501 ± 0.899 (3.038–3.963)	+4.5% *p* = 0.057	47%
Cumulated mannitol dose (mg)	415 ± 223 (300–530)		550 ± 171 (462–638)	+32.5% *p* = 0.023	65%
Maximal percent FEV_1_ decrease (%)	15.4 ± 7.1 (11.7–19.0)		9.8 ± 8.1 (5.6–14.0)	−36.4% *p* = 0.027	62%
PD15 FEV_1_	308 ± 169 (187–428)		282 ± 196 (−29 to 594)	−9.2% *p* = 0.989	5%
Antilog (base 10) PD15 FEV_1_	207 ± 4 (79–548)		232 ± 2 (72–745)	+12.1% *p* = 0.590	5%
RDR mannitol	0.13 ± 0.32 (−0.03 to 0.29)		0.03 ± 0.04 (0.02–0.05)	−76.9% *p* = 0.099	23%
Antilog (base 10) RDR mannitol	0.04 ± 4.41 (0.02–0.09)		0.02 ± 3.20 (0.01–0.04)	−50% *p* = 0.024	65%

FEV_1_: forced expiratory volume in one second. PD15 FEV_1_: mannitol dose leading to reduction in FEV_1_ during a bronchial provocation test of ≥15%. RDR: response dose ratio is the percent decrease in FEV_1_ at the end of challenge divided by the cumulative dose of mannitol causing decrease in FEV_1_.

**Table 3.** Changes in methacholine test. Mean values ± SD (95% confidence interval).

	Baseline visit	Interim visit (+2 weeks)	Final visit (+6 weeks)	% change final vs. baseline (*p*-value)	Power for 5% significance
FEV_1_ (L)	3.316 ± 0.895 (2.839–3.793)	3.634 ± 1.057 (3.090–4.177)	3.397 ± 0.789 (2.992–3.803)	+2.4% *p* = 0.340	7%
Cumulated methacholine dose (mg)	3.26 ± 1.99 (2.20–4.32)		3.82 ± 1.58 (3.00–4.63)	+17.2% *p* = 0.237	19%
Maximal percent FEV_1_ decrease (%)	23.4 ± 10.8 (17.9–29.0)		22.2 ± 10.7 (16.7–27.7)	−5.1% *p* = 0.352	6%
PD20 FEV_1_	1.80 ± 1.15 (1.02–2.57)		2.42 ± 1.17 (1.34–3.50)	+34.4% *p* = 0.174	17%
Antilog (base 10) PD20 FEV_1_	1.48 ± 1.97 (0.94–2.33)		2.19 ± 1.62 (1.41–3.43)	+48.0% *p* = 0.129	22%
RDR methacholine	13.1 ± 11.8 (6.8–19.4)		6.8 ± 5.6 (3.9–9.7)	−48.1% *p* = 0.01	78%
Antilog (base 10) RDR methacholine	8.40 ± 2.94 (4.73–14.93)		4.68 ± 2.69 (2.81–7.76)	−44.3% *p* = 0.006	84%

FEV_1_: forced expiratory volume in one second. PD20 FEV_1_: methacholine dose leading to reduction in FEV_1_ during a bronchial provocation test of ≥20%. RDR: response dose ratio is the percent decrease in FEV_1_ at the end of challenge divided by the cumulative dose of methacholine causing decrease in FEV_1_.

**Table 4.** Changes in inflammation parameters. Mean values ± SD (95% confidence interval).

	Baseline visit	Interim visit (+2 weeks)	Final visit (+6 weeks)	% change final vs. baseline (*p*-value)
Blood eosinophil count (G/L)	0.26 ± 0.21 (0.15–0.37)		0.21 ± 0.20 (0.11–0.31)	−19.2% *p* = 0.218
Eosinophil cationic Protein (µg/L)	16.9 ± 8.8 (12.3–21.6)		18.4 ± 12.9 (11.5–25.2)	+8.9% *p* = 0.206
NO	31.6 ± 23.2 (19.7–43.5)	20.0 ± 13.4 (12.5–27.4)	21.5 ± 12.2 (15.1–28.0)	−32.0% *p* = 0.0784
Antilog (base 10) NO	24.9 ± 2.1 (17.1–36.1)	16.5 ± 1.9 (11.6–23.4)	18.4 ± 1.8 (13.3–25.4)	−26.1% *p* = 0.068

Changes of blood eosinophil counts, serum ECPs, and FeNO were consistent with the results obtained by the provocation tests. Differences between baseline and final visit did not reach statistical significance; all numerical trends were consistently in favor of a possible modest improvement of airway inflammation (Table 4).

Asthma-related quality of life assessed by the AQLQ improved significantly in all four domains covered by the Juniper questionnaire (Table 5). The overall quality of life increased significantly from a high baseline value of 5.3 to 6.1 between baseline and the final visit (+15.1%, *p* < 0.001). In addition, 11 patients (65%) experienced a clinically relevant improvement in asthma-related quality of life (>0.5 improvement in score).

**Table 5.** Changes in asthma-related quality of life by subcategories according to Juniper (numerical scale from 1 to 7; 1 severely impaired, 7 not impaired at all). Mean values ± SD (95% confidence interval).

	Baseline visit	Interim visit (+2 weeks)	Final visit (+6 weeks)	% change final vs. baseline (*p*-value)
Overall	5.3 ± 1.1 (4.7–5.9)	5.6 ± 1.3 (5.0–6.3)	6.1 ± 1.0 (5.6–6.6)	+15.1% *p* < 0.001
Daily activities	5.5 ± 1.0 (5.0–6.0)	5.7 ± 1.2 (5.1–6.3)	6.1 ± 1.0 (5.6–6.6)	+10.9% *p* < 0.001
Asthma symptoms	5.0 ± 1.4 (4.3–5.7)	5.5 ± 1.5 (4.7–6.3)	6.0 ± 1.0 (5.5–6.5)	+20.0% *p* < 0.001
Emotional life	5.5 ± 1.3 (4.8–6.1)	5.8 ± 1.4 (5.0–6.5)	6.1 ± 1.2 (5.5–6.7)	+11.2% *p* = 0.001
Environmental exposure	5.4 ± 1.4 (4.7–6.2)	5.9 ± 1.3 (5.3–6.5)	6.1 ± 1.2 (5.4–6.7)	+13.0% *p* = 0.002

## Discussion

In this pilot study with 17 well-controlled asthma patients, short-term treatment with montelukast added to a basis therapy with ICS + LABA for 4 weeks improved all parameters of airway hyperresponsiveness and inflammation in a consistent manner, suggesting that, even in clinically well-controlled asthmatics, residual inflammation amenable to therapeutic intervention may persist.

Airway hyperresponsiveness is a hallmark of asthma that can be assessed by provocation tests using direct and/or indirect stimuli^28^. Indirect challenge tests may be a closer reflection of active airway inflammation than direct challenge tests^29,30^. While direct challenge tests (such as the methacholine test) act directly on the airway smooth muscle, indirect challenge tests (such as the mannitol test) act by increasing the osmolarity of the airway surface liquid which leads to the release of mediators from inflammatory cells (such as prostaglandins, leukotrienes, and histamine) and of neuropeptides from sensory nerves. Ultimately, in subjects with bronchial hyperresponsiveness this cascade of events leads to airway narrowing and reduction of FEV_1_. Thus, as most stimuli that provoke an attack of asthma in daily life do so indirectly, indirect tests may be considered more specific for asthma^17^. The present study showed improvements in airway hyperresponsiveness when using an indirect challenge test with mannitol. A positive test to mannitol was shown to correlate with asthma severity, asthma worsening during stepwise reduction of ICS, asthma symptoms, and asthma airway inflammation consistent with the presence of inflammatory cells such as eosinophils or their mediators such as prostaglandins, leukotrienes, and histamine^31–33^. Earlier observations suggested that leukotriene receptor antagonists play a key role in sustaining but not in initiating the airway response to mannitol^34,35^, which is consistent with our observations. On the other hand, a negative result may be due to an insufficient number of inflammatory cells (as it may occur in a patient on ICS), insufficient concentration of mediators or lack of airway hyperresponsiveness to mediators (as it may occur in nonasthmatic patients with eosinophilic bronchitis)^36^. Keeping this limitation in mind, the mannitol test is useful to confirm diagnosis of a currently active asthma in untreated or of insufficiently controlled asthma in treated patients^30,31,36^. In the present study, montelukast added to ICS + LABA over 4 weeks significantly improved bronchial reactivity and lead to a significant increase of the cumulated dose of mannitol administered. On the other hand, montelukast had no effect on airway sensitivity assessed by the mannitol PD15. Thus, the improvements reported in the present study suggest that montelukast might possibly contribute to additionally decrease airway hyperresponsiveness when added to a fixed combination of ICS + LABA even in already well-controlled patients. Bronchial provocation testing with methacholine is generally considered sensitive, i.e. useful to rule out the presence of asthma in a person not taking ICS^37^. However, 27% of adults reporting physician diagnosed asthma, generally with normal or near normal spirometry, were shown to have a negative methacholine challenge test^38^. On the other hand, a positive methacholine provocation test does not necessarily rule in the diagnosis of asthma^31^ so that airway hyperresponsiveness has been described in patients with allergic rhinitis^39^ and with chronic obstructive pulmonary disease^40^. Sensitivity to the test is expressed as the PD20, i.e. the cumulated provocative dose of methacholine (in mg) inducing a 20% fall in FEV_1_. Reactivity is characterized by the response–dose ratio (RDR), i.e. the final % fall in FEV_1_ divided by the total cumulative dose needed to induce that % fall in FEV_1_^41^. The latter was first suggested as a simple index of non-specific airway reactivity summarizing the dose–response curve by O’Connor *et al*.^42^ and applied to the findings of the Swiss SAPALDIA study to establish reference values for methacholine reactivity^43^. In the present study, airway reactivity assessed by the methacholine challenge significantly improved after 4 weeks of add-on therapy with montelukast compared to baseline, which is consistent with previous findings in patients with asthma^10,44–46^. However, airway sensitivity only numerically improved by up to 48% without reaching statistical significance. These observations support previously reported additional protection against excessive airway narrowing provided by montelukast added to a stable dose ICS in a 12 weeks’ placebo controlled study in patients with controlled asthma. In this study, montelukast significantly improved the FEV_1_ decline and increased PD20 FEV_1_ vs. placebo, thus providing additional protection to ICS with regard to excessive airway narrowing^47^.

Although their contribution to asthma management remains unclear, non-invasive markers of airway inflammation, such as the levels of exhaled nitric oxide^48^ and the number of eosinophils in the blood or the sputum, may be of interest to assess the level of inflammation underlying the current asthma status, guide treatment choice, and monitor treatment response^49^. Eosinophils present in increased numbers in airways release proteins that may damage airway epithelial cells. They also play a role in the release of growth factors and airway remodeling^50^. Cysteinyl leukotrienes are potent proinflammatory mediators mainly derived from eosinophils and mast cells. They are the only mediator whose inhibition has been associated with an improvement in lung function and asthma symptoms^51^. Furthermore, there is increasing evidence for the involvement of cysteinyl leukotrienes in the pathophysiology of airway hyperresponsiveness and, subsequently, in airway remodeling^52–54^. Thus, the trend in numerical decrease of blood eosinophils observed in the present study is consistent with previously published findings showing that montelukast decreases the number of eosinophils and mast cells in airway tissues^55^ and the blood of asthma patients^56^.

Nitric oxide (NO) is produced by many cells, including eosinophils. NO can act as a dilator of bronchial and vascular smooth muscle, a neurotransmitter and an immune response mediator^57^. Although normal ranges are still to be defined^58^, NO can be measured in the exhaled air with non-invasive standardized methods^59^ allowing for longitudinal monitoring. Exhaled NO can be considered a surrogate marker of airway inflammation which increases with loss of asthma control^60,61^ and decreases with improving inflammation control^62^. In our study, exhaled NO levels decreased markedly and early (2 weeks) after initiation of the add-on therapy with montelukast, which is consistent with improved control of airway inflammation, especially when taken together with the effects on the other non-invasive markers of inflammation. It is well-known that ICS dose-tapering in well-controlled patients may lead to loss of asthma control. Airway hyperresponsiveness to mannitol was identified as a predictor of asthma exacerbations after ICS dose reduction^32^ so that responsiveness to mannitol can be used to predict risk of exacerbation during back titration of steroids^63^. On the other hand, airway hyperresponsiveness correlates with noninvasive markers of airway inflammation such as exhaled nitric oxide and sputum eosinophils in steroid-naive asthmatics only^33^. Thus the incremental response of montelukast added to ICS + LABA observed in the present study may suggest that montelukast contributes to improved asthma control via improved control of airway inflammation which is consistent with earlier reports indicating that leukotriene receptor antagonists oppose systemic mediators of inflammation which are not controlled by or not accessible to inhaled corticosteroids^64^.

Quality of life improvements observed in the present study were statistically significant in all domains and clinically relevant in two-thirds of the patients. The determinants of quality of life in asthma are not fully understood. Female sex, the presence of respiratory symptoms, being a current or former smoker, and the presence of an associated allergic rhinitis were independently associated with poorer quality of life in asthmatics^65–67^. In the present study, the majorities of patients included were women, had no symptoms, and were non-smokers. A potential confounder was the presence or absence of allergic rhinitis which was not assessed at inclusion. A future study should aim at also characterizing such determinants of quality of life in asthmatics not covered by the Juniper questionnaire, e.g. for a stratified analysis. Keeping in mind that the present study had no control group, but given the fact that patients were well-controlled at inclusion, this suggests that montelukast may contribute to improve quality of life in asthmatics by still unknown mechanisms beyond inflammation control and lung function improvement.

This pilot study has several limitations. First, this study had no control group such that reported statistical significances relate to differences versus baseline. A future study should ideally include a placebo control group. Furthermore, only patients with well-controlled asthma on ICS and LABA were included. A future study may want to explore the effects of montelukast added to ICS alone and use patients on ICS alone or on ICS plus LABA as a control group. The number of patients included in this pilot study was too small for assessing airway sensitivity but deemed sufficient for assessing airway reactivity. The present results should allow for adequate power calculations, depending on the primary endpoint chosen. Finally, all comparisons were made versus baseline, that is before the 2-week run-in on a standardized ICS + LABA regimen. In a future study, it would be advisable to re-assess the primary outcomes following run-in.

In this small cohort of 17 patients consistent trends across all tested dimensions of airway hyperresponsiveness and inflammation suggest that in an adequately powered study, the add-on effects of montelukast added to a basis therapy with ICS + LABA may contribute to improved asthma control. The present results may contribute to facilitate power and thus sample size calculations in future clinical trials aimed at exploring more in depth the effects of montelukast on airway inflammation and hyperresponsiveness.

## Conclusion

Montelukast added to a fixed combination of ICS + LABA in well-controlled asthma patients was shown to numerically improve airway hyperresponsiveness and selected parameters of airway inflammation. While statistical significance was not reached in this pilot study, the consistency of the reported numerical trends across all parameters of interest deserves further research in adequately designed and powered randomized controlled trials.

## Transparency

### Declaration of financial/other relationships

P.C. is a paid consultant/advisor for CIPLA Ltd. A.H. served as an advisory board member for Merck. None of the other authors declare any financial relationship or conflict of interest.
